# Determination of Heavy Metals in Groundwater Around Al-Buraihi Sewage Station in Taiz City, Yemen

**DOI:** 10.5696/2156-9614-11.30.210604

**Published:** 2021-06-17

**Authors:** Raya Qaid Alansi, Abdelhafeez M.A. Mohammed, Mahmoud M. Ali, Wadie Ahmed Mokbel Ghalib, Sajan Chimmikuttanda Ponnappa

**Affiliations:** 1 Department of Applied Chemistry, Faculty of Applied Science, International University of Africa, Khartoum, Sudan; 2 Department of Chemistry, Rabigh College of Science & Arts, King Abdulaziz University, Rabigh, Saudi Arabia; 3 Taiz University, Faculty of Medical and Health Sciences, Laboratories Department-Taiz, Yemen; 4 VerdeEn Chemicals Pvt. Ltd, Hapur District, Uttar Pradesh, India

**Keywords:** pollution, contamination, groundwater, ICP-OES

## Abstract

**Background.:**

In recent years, mitigation of groundwater contamination resulting from the limited availability of freshwater for domestic use has become an important issue. The presence of heavy metals in water could have adverse effects on both plant and animal life.

**Objectives.:**

The main objective of the present study was to determine possible heavy metal contamination in groundwater around Al-Buraihi sewage station in Taiz, Yemen and to understand possible sources of contamination and their relationships with groundwater.

**Methods.:**

Wastewater samples were collected from a wastewater stabilization pond from Al-Buraihi sewage station and borewell water samples were collected from the vicinity. The presence of heavy metals was quantified using inductively coupled plasma-optical emission spectrometry (ICP-OES). Pearson correlation test was performed to understand the relationship between wastewater and groundwater samples.

**Results.:**

Physical variables including pH, electrical conductivity (EC), dissolved oxygen (DO) and temperature and elements such as silver (Ag), arsenic (As), aluminum (Al), barium (Ba), boron (B), cadmium (Cd), chromium (Cr), iron (Fe), molybdenum, nickel (Ni), selenium (Se) and zinc (Zn) exceeded the permissible limits recommended by international standards in wastewater samples.

**Conclusions.:**

Treated sewage wastewater in the study area is not suitable for irrigation as the elements/heavy metals are accumulated in soil and plants and may be accumulated in humans and animals through bio-accumulation. In addition, these heavy metals reach the water table and aquifers through percolation, thereby polluting groundwater.

**Competing Interests.:**

The authors declare no competing financial interests.

## Introduction

The multiparty war in Yemen has continued for the past six years, affecting millions of people.^1^ In the Taiz Governorate, civilians continue to bear the brunt of the conflict. About 3 million people live in the Taiz Governorate, which accounts for approximately 11.3 percent of the country's population.^2^ Since the conflict began in 2015, Taiz— especially Taiz city, the governorate's capital—has been a hotspot for fighting, with all parties involved reporting violations of international humanitarian law. Long-term heavy artillery exchanges, as well as indiscriminate bombing, sniper fire, rocket attacks, the use of landmines, and airstrikes, have taken place in and around Taiz, even in residential areas.^3^ Water is one of many commodities that is rapidly depleting in Yemen, sometimes related to the war.

Clean water is essential for human health and quality of life.^4^ Population expansion has led to an increase in demand for resources which has ultimately led to a global rise in industrialization and urbanization.^5^ This has increased the demand for freshwater, which has been exploited more than any other resource. The overutilization of water resources has deteriorated the quality of freshwater through contamination and pollution.^6^

Water contamination is presence of chemicals/foreign materials/substances out of place and/or present at a higher concentrations than normal concentrations that have adverse effects on any non-targeted organism.^7^ To overcome this problem, water treatment is required.^8^ Wastewater treatment removes pollutants from wastewater in order to reuse treated water for other activities.^9^–^11^ The water crisis faced by many countries is the main cause of the increase in the reuse of treated wastewater worldwide.^12^ In arid and semi-arid regions, water resource planning involves the reclamation and reuse of wastewater.^13^–^16^ However, improper maintenance of wastewater treatment ponds, improper lining or overutilization of wastewater without proper treatment for agricultural practices can lead to the pollution of soil and groundwater. Soil and groundwater may contain pollutants such as heavy metals which can eventually enter the food chain.^17^ Toxic heavy metals are harmful to human health and present a threat to both plant and animal life.^18^–^23^ Monitoring of heavy metals in environmental samples is crucial since most of these heavy metals can influence human health (positively or negatively) even at very low concentrations.^24^–^26^ The present study aimed to determine the level of heavy metal contamination in the wastewater treatment pond around Al-Buraihi sewage station in Taiz city, Yemen.

**Abbreviations**
*EC*: Electrical conductivity

## Methods

**Figure 1:**
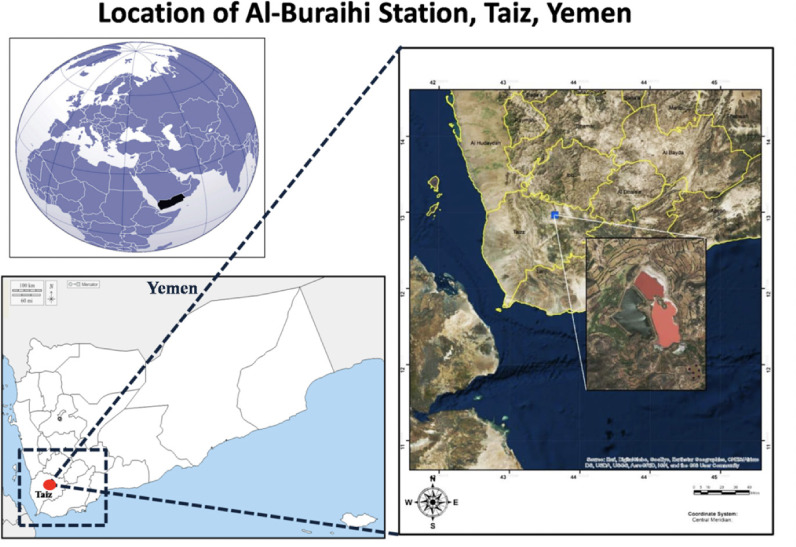
Study Area

Al-Buraihi sewage station in Taiz city is located in the Al-Buraihi area, to the northeast of Taiz. The upper and lower altitudes of this area consist of a preliminary processing unit. Pond 1 is located at higher altitude while ponds 2 and 3 at a lower altitude. The sewage station is located between longitudes 10° 39′ and 30° 39′ N and latitudes 80° 150′ and 80° 151′ E. The climatic condition of Al-Buraihi area is warm and semi-dry, with an average annual temperature of 25°C. Al-Buraihi station consists of four basins as shown in Figure 1. The first and the second basin have an area of 18000 m^2^, with a depth of 4.25 m. These basins operate alternatively at an interval of four years each. They are anaerobic ponds. The third basin has an area of 248800 m^2^ and 5.3 m depth. This is a facultative pond and the fourth basin has an area of 146800 m^2^ and 5.3 m depth and is a maturation pond. None of the four ponds has a concrete lining. Before the construction of Al-Buraihi wastewater station, this area was a swampy region with few wells.

### Materials

All chemicals and reagents used in the present study were of analytical grade (AR) nitric acid (65% American Chemical Society (ACS)), International Standards Organization (ISO)), perchloric acid (HClO_4_) (70% ACS, ISO) and sulfuric acid (H_2_SO_4_) (98%–VWR) extra pure were used. Standard solutions of salts of elements (1000 mg/L) were purchased from Scharlau, Spain. All glassware was soaked in 10% nitric acid and washed with Millipore distilled water before use.

### Instrumentation

An inductively coupled plasma-optical emission spectrometer (ICP-OES) with an axially viewed configuration (VISTA MPX, Varian, Mulgrave, Australia) equipped with a solid-state detector, Stumar-master mist chamber, and V-groove nebulizer was employed for element determinations using a standard calibration method. Electrical conductivity (EC) and pH were determined *in-situ* using a multipurpose electronic Jenway 4520 conductivity/total dissolved solids (TDS) meter and Hanna portable pH meter, respectively. The dissolved oxygen concentration of wastewater samples was measured immediately in the field by using Inolabmulti 720, (Willis Towers Watson (WTW)).

### Wastewater and groundwater sampling

**Figure 2:**
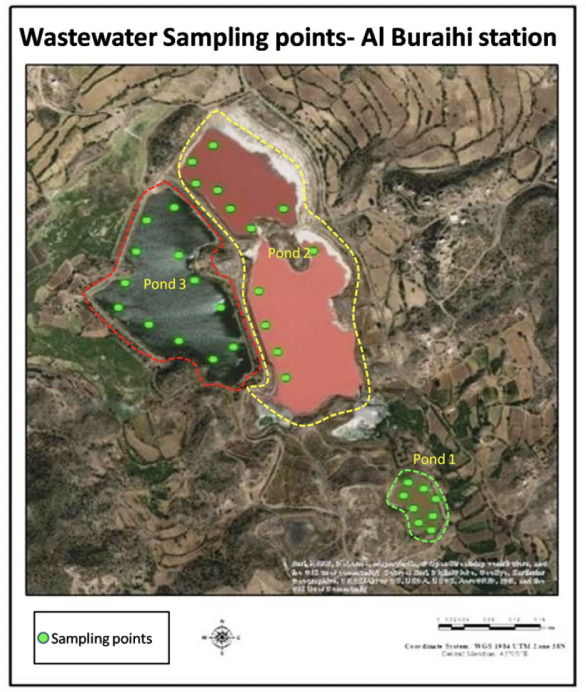
Wastewater sampling points, Al Buraihi station

**Figure 3:**
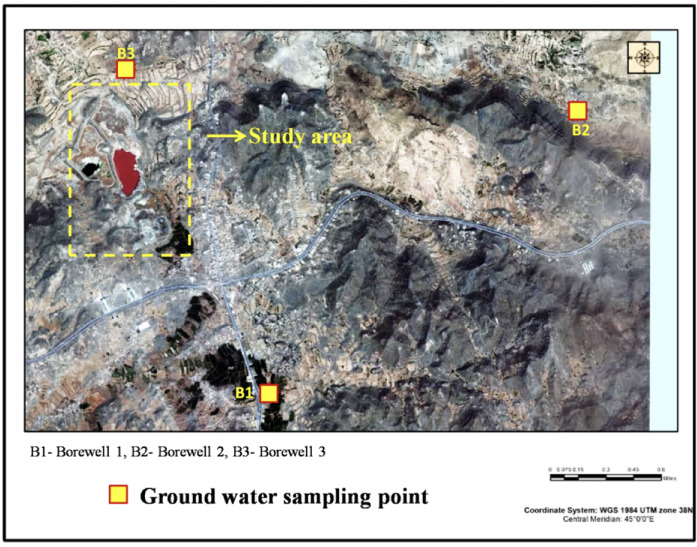
Groundwater sampling points

**Figure 4:**
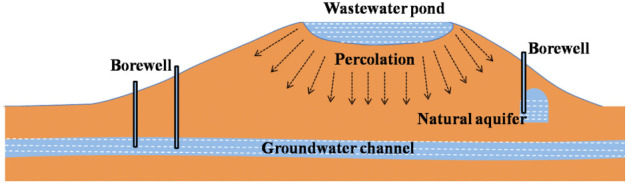
Schematic sketch of the study area showing the possibilities of wastewater percolation reaching groundwater (channel and aquifer)

Water sample collection for the present study was performed during the summer season in 2017 (July to September). Thirty-two wastewater samples were taken from three positions at different times to determine heavy metal concentrations in wastewater stabilizing ponds in Al-Buraihi *(Figure 2).* Groundwater samples were also collected from the borewells located around the proximity of the wastewater station *(Figure 3).* It should be noted that the wastewater station is located in a geographically higher region compared to the borewells from which the groundwater samples were collected. The collected samples were transported in an ice box to be kept under ambient temperature until analysis. Wastewater samples were stored in a fridge at approximately 4°C. All samples were acidified at the time of collection with nitric acid (HNO_3_) (5mL) to prevent microbial degradation of heavy metals and to ensure sterility. All plastic containers for samples were prewashed with distilled water before being used.^27^ A schematic diagram of the study area is presented in Figure 4.

### Sample Preparation

Wastewater samples were pretreated with concentrated HNO_3_ to prevent microbial degradation of heavy metals. Then, 10 mL of wastewater samples were digested by adding 7.0 mL of HNO_3_ (65%), 3.0 ml hydrogen peroxide (H_2_O_2_) (30%) and diluted to 50 mL with distilled water. All reagents were of analytical grade (AR) and purchased from (Scharlau-JPN) including standard stock solutions of known concentration of different heavy metals. All analyses were done in triplicate. The analytical procedure followed for these tests were the procedures given in the operation manuals of the instrument used and by the American Public Health Association (APHA) 1999.^28^

### Statistical analysis

Statistical Program for the Social Sciences (SPSS) version 26 software was used to calculate the descriptive statistics of mean, standard deviation, and correlation analysis. The data collected were discussed in terms of average and 95% confidence intervals. Statistical differences between means were compared using the least significant differences (LSD) with a P value ≤ 0.05 indicating significance.

## Results

Wastewater and borewell water samples were subjected to ICPOES analysis. The results were compared with the water quality standards obtained from World Health Organization (WHO),^29^ Yemen Standardization, Metrology and Quality Control Organization (YSMO),^30^ Joint Food and Agriculture Organization of the United Nations (FAO)/WHO Expert Committee on Food Additives (JECFA),^31^ and Indian Standard for Drinking Water as per Bureau of Indian Standards (BIS) specifications (IS 10500-2012).^32^ The water quality standards are given in Table 1. In total, 32 wastewater samples were collected from three wastewater stabilizing ponds depending on their size. Eight samples were collected from pond 1. Twelve samples each were collected from ponds 2 and 3. The collected samples were analyzed using ICP-OES. Descriptive statistics of the physicochemical properties of wastewater samples collected from the study area are given in Table 2. Since there were only three borewells in proximity to the study area, groundwater samples were collected from these borewells. The location, depth, and physicochemical properties of borewell water samples are given in Table 3. The samples collected were designated as B1, B2, and B3. A graphical representation of the physicochemical variables of the wastewater samples is given in Figure 5. A graphical representation of the physicochemical variables of the borewell samples is given in Figure 6.

**Table 1:** Water Quality Standards for Wastewater and Drinking Water

**Variable**	**Wastewater**		**Drinking water**	
Ag	0.010^a^			0.1^d^
Al	2^a^	5^b^	0.9^c^	0.03
As	0.05^a^	0.1^b^	0.01^c^	0.01^d^
B	1.0^a^	2- 0.7^b^	2.4^c^	0.5^d^
Ba	1^a^		0.7^c^	0.7^d^
Be	0.1^a^	0.1^b^	0.01^c^	
Cd	0.01^a^	0.01^b^	0.003^c^	
Co	0.05 a	0.05^b^		
Cr	0.02 a	0.1^b^	0.05^c^	0.05^d^
Cu	0.2 a	0.2^b^	2^c^	
Fe	5^a^	5^b^		0.3^d^
Li	2.5^a^	5^b^		
Mn	0.2^a^	0.2^b^		0.1^d^
Mo	0.01^a^	0.01^b^		0.07^d^
Ni	0.2^a^	0.5^b^	0.07^c^	0.02^d^
Pb	0.2^a^	5^b^	0.01^c^	0.01^d^
Sb			0.02^c^	
Se	0.05^a^	0.02^b^	0.04^c^	0.01^d^
Sn	10^a^			
Sc				
Zn	5^a^	2^b^		5d
Sr				
K				
Mg	60 a			30^d^
Na	200^a^	200^b^		
Ca	200^a^			75^d^
P	30^a^			
EC (dS/m)		0.7–4^b^	0.399	
pH	6.5–8.4 a	8.4–6.5^b^		6.5–8.5^d^
DO	>2.0^a^	2^b^		
OM (%)				
Temp(°C)				
SAR		40^b^		

Abbreviations: EC, electrical conductivity; DO, dissolved oxygen; OM, organic matt Temp, temperature; SAR, sodium absorption ratio.

a WHO^29^

b Yemen Standardization, Metrology and Quality Control Organization^30^

c Joint FAO/WHO Expert Committee on Food Additives (JECFA)^31^

d BIS Bureau of Indian Standards? (Yes)(10500-2012) water quality standards.^32^

**Table 2:** Descriptive Statistics of Physicochemical Properties of Wastewater Samples Collected from the Study Area

	**Variables**	**Unit**	**Minimum**	**Maximum**	**Mean**	**SD**
Pond 1 (n=8) mg/L	pH	-	7.6	8.4	8.075	0.255
	EC	dS/m	6.0	7.2	6.463	0.459
	DO	mg/L	0.3	2.1	0.750	0.748
	Temp.	°C	28.3	37.8	31.563	3.967
Pond 2 (n=12) mg/L	pH	-	8.1	8.7	8.325	0.171
	EC	dS/m	5.4	7.8	6.325	0.637
	DO	mg/L	0.2	6.2	1.608	1.778
	Temp.	°C	24.0	33.4	28.758	2.753
Pond 3 (n=12) mg/L	pH	-	8.6	8.9	8.750	0.079
	EC	dS/m	5.7	7.6	6.950	0.552
	DO	mg/L	1.4	13.9	5.617	4.309
	Temp.	°C	22.8	35.3	28.625	4.332

**Table 3:** Physicochemical Properties of Groundwater Samples Collected from Study Area

Borewell	**Depth (m)**	**Locations**		**Elevation (m)**	**pH**	**EC μS/cm or ds/m**	**Temp (°C)**	**DO_2_ (mg/l)**	**Placement**

		Areax	Areay						
B1	17	391603	1511077	1141	7.8	5590/5.59	28.9	3.3	Open bore well
B2	12	392856	1508260	1177	8	7880/7.88	27.2	2	Close to manhole of wastewater used Animals drinking water
B3	14	395541	1510717	1183	7.43	7180/7.18	28.1	3.4	

Abbreviations:EC, electrical conductivity; DO, dissolved oxygen; Temp, temperature; B1, Borewell 1; B2, Borewell 2; B3, Borewell 3.

Descriptive statistical values of all the elements including heavy metals for the wastewater samples collected from the three ponds are given in Table 4. For comparison, the mean value of the elements for the wastewater samples is included with the results of the borewell water samples in Table 5. To understand the relationship between wastewater samples and groundwater samples, Pearson correlation analysis of wastewater and borewell water samples was performed. The results are given in Table 6.

**Table 4:** Descriptive Statistics of Elements in Water Samples Collected from Wastewater Ponds

	**Pond I (n =8) mg/L**				**Pond II (n =12) mg/L**				**Pond III (n =12) mg/L**			
	Min	Max	Mean	SD	Min	Max	Mean	SD	Min	Max	Mean	SD
Ca	77.99	509.02	348.8	182.3	36.74	426.35	290.4	130.49	31.201	378.04	208.00	115.02
K	30.35	88.899	64.80	18.30	42.39	88.405	62.66	11.85	45.104	94.849	69.233	12.672
Mg	109.72	567.59	408.9	198.9	166.89	734.41	518.8	187.4	156.23	878.94	604.35	280.37
Na	404.12	891.23	709.5	154.2	633.4	1162.4	840.81	147.4	748.00	1239.9	974.38	135.59
P	12.85	81.527	34.3	24.6	12.58	59.649	24.084	14.5	5.287	23.345	13.932	6.38
Ag	0.005	38.892	11.115	15.6	0.011	23.031	3.339	6.79	0.013	25.412	4.855	9.4023
Al	0.000	0.857	0.506	0.278	0.000	0.744	0.397	0.29	0.000	4.097	0.555	1.152
As	0.000	0.115	0.043	0.041	0.000	0.084	0.02	0.03	0.000	0.068	0.019	0.027
B	1.042	1.317	1.198	0.086	1.209	2.088	1.44	0.24	1.366	2.215	1.838	0.246
Ba	0.000	8.826	1.213	3.079	0.000	0.204	0.03	0.05	0.000	0.111	0.019	0.029
Be	0.000	0.000	0.000	0.000	0.000	0.000	0.000	0.00	0.000	0.001	0.000	0.000
Cd	0.000	0.084	0.019	0.029	0.000	0.021	0.004	0.00	0.000	0.102	0.011	0.029
Co	0.000	0.015	0.007	0.004	0.000	0.048	0.01	0.011	0.000	0.035	0.012	0.009
Cr	0.000	5.403	0.691	1.904	0.002	1.552	0.14	0.443	0.000	9.641	0.823	2.777
Cu	0.013	0.071	0.040	0.025	0.000	0.100	0.026	0.028	0.000	0.252	0.039	0.077
Fe	0.000	2.781	0.754	0.866	0.000	6.718	0.863	1.849	0.160	8.411	1.435	2.399
Li	0.067	0.084	0.074	0.007	0.053	0.081	0.069	0.009	0.037	0.084	0.069	0.014
Mn	0.046	0.196	0.107	0.048	0.016	0.252	0.079	0.062	0.001	0.157	0.045	0.041
Mo	0.000	0.028	0.011	0.009	0.000	0.118	0.019	0.034	0.000	0.046	0.012	0.016
Ni	0.000	0.107	0.033	0.033	0.000	1.497	0.151	0.425	0.000	0.085	0.027	0.026
Pb	0.011	0.103	0.045	0.033	0.014	0.051	0.033	0.010	0.004	0.107	0.043	0.031
Sb	0.000	0.078	0.029	0.032	0.000	0.078	0.020	0.026	0.000	0.084	0.014	0.024
Se	0.000	0.072	0.029	0.032	0.000	0.230	0.036	0.069	0.000	0.138	0.026	0.043
Sn	0.000	0.203	0.078	0.081	0.000	0.220	0.049	0.067	0.000	0.603	0.116	0.219
Zn	0.014	0.379	0.145	0.115	0.063	11.796	1.242	3.342	0.016	2.521	0.693	0.906

**Table 5:** Elements Present in Wastewater Ponds and Borewell Water

	**Mean values of variables of pond samples mg/L**			**Borewell water sample variables mg/L**		
	**Pond 1**	**Pond 2**	**Pond 3**	**B1**	**B2**	**B3**
Ca	348.863	290.414	208.000	228.26	164.34	186.56
K	64.802	62.661	69.233	28.54	20.88	24.08
Mg	408.943	518.899	604.352	156.92	144.36	148.88
Na	709.591	840.871	974.381	289.45	236.32	246.72
P	34.354	24.084	13.932	15.46	8.84	12.334
Ag	11.115	3.339	4.855	1.24	0.60	0. 72
Al	0.506	0.397	0.555	0.12	0.08	0.08
As	0.043	0.027	0.019	0.004	0.002	0.002
B	1.198	1.443	1.838	0.068	0.035	0.042
Ba	1.213	0.039	0.019	0.006	0.006	0.004
Be	0.000	0.000	0.000	0.000	0.000	0.000
Cd	0.019	0.004	0.011	0.002	0.001	0.001
Co	0.007	0.014	0.012	0.004	0.002	0.002
Cr	0.691	0.147	0.823	0.06	0.02	0.03
Cu	0.040	0.026	0.039	0.02	0.02	0.03
Fe	0.754	0.863	1.435	0.36	0.24	0.28
Li	0.074	0.069	0.069	0.004	0.002	0.001
Mn	0.107	0.079	0.045	0.08	0.03	0.05
Mo	0.011	0.019	0.012	0.006	0.002	0.001
Ni	0.033	0.151	0.027	0.004	0.002	0.002
Pb	0.045	0.033	0.043	0.02	0.01	0.01
Sb	0.029	0.020	0.014	0.008	0.004	0.003
Se	0.029	0.036	0.026	0.002	0.000	0.000
Sn	0.078	0.049	0.116	0.002	0.001	0.001
Zn	0.145	1.242	0.693	0.067	0.032	0.048

**Table 6:** Pearson Correlation Analysis of Wastewater Samples and Borewell Water Samples

	Ca	K	Mg	Na	P	Ag	Al	As	B	Ba	Be	Cd	Co	Cr	Cu	Fe	Li	Mn	Mo	Ni	Pb	Sb	Se	Sn	Zn
Ca	1																								
K	0.685	1																							
Mg	0.488	.963**	1																						
Na	0.534	.977**	.998**	1																					
P	.993**	0.667	0.461	0.507	1																				
Ag	.840*	0.757	0.57	0.613	.876*	1																			
Al	0.644	.987**	.943**	.959**	0.638	0.799	1																		
As	.922**	.859*	0.708	0.743	.934**	.936**	.850*	1																	
B	0.526	.977**	.997**	.999**	0.504	0.629	.965**	0.748	1																
Ba	0.798	0.446	0.207	0.258	.855*	.917*	0.495	0.803	0.277	1															
Be	.c	.c	.c	.c	.c	.c	.c	.c	.c	.c	.c														
Cd	0.735	0.769	0.605	0.644	0.773	.981**	.833*	.877*	0.664	.861*	.c	1													
Co	0.487	.887*	.946**	.940**	0.429	0.395	.820*	0.623	.925**	0.041	.c	0.392	1												
Cr	0.448	.826*	0.766	0.786	0.471	0.797	.903*	0.694	0.805	0.541	.c	.890*	0.536	1											
Cu	0.475	0.746	0.664	0.681	0.528	0.792	0.806	0.694	0.706	0.604	.c	.854*	0.421	.896*	1										
Fe	0.32	.903*	.958**	.954**	0.294	0.506	.914*	0.569	.956**	0.121	.c	0.592	.858*	.841*	0.699	1									
Li	0.735	.989**	.940**	.955**	0.724	0.791	.973**	.906*	.957**	0.511	.c	0.783	.867*	0.79	0.727	.846*	1								
Mn	.932**	0.466	0.241	0.296	.913*	0.699	0.42	0.737	0.277	0.73	.c	0.59	0.278	0.273	0.306	0.11	0.494	1							
Mo	0.679	.869*	.868*	.875*	0.618	0.49	0.787	0.735	.855*	0.205	.c	0.44	.957**	0.455	0.345	0.725	.875*	0.499	1						
Ni	0.526	0.581	0.62	0.612	0.468	0.182	0.454	0.517	0.588	.0.004	.c	0.073	0.805	0.028	0.036	0.398	0.624	0.352	.875*	1					
Pb	0.735	.966**	.882*	.909*	0.722	.852*	.979**	.881*	.910*	0.58	.c	.875*	0.77	.892*	0.772	.854*	.950**	0.565	0.779	0.412	1				
Sb	.950**	.847*	0.688	0.726	.949**	.920**	.831*	.992**	0.726	0.791	.c	.855*	0.629	0.66	0.627	0.548	.890**	0.793	0.761	0.532	.883*	1			
Se	0.751	.957**	.922**	.934**	0.726	0.696	.909*	.879*	.929**	0.422	.c	0.658	.916*	0.645	0.595	0.786	.977*	0.516	.947**	0.775	.884*	.872*	1		
Sn	0.454	.931**	.924**	.932**	0.455	0.714	.970**	0.715	.944**	0.385	.c	0.792	0.758	.952**	.839*	.956**	.896*	0.229	0.661	0.293	.927**	0.683	0.799	1	
Zn	0.323	0.687	0.798	0.778	0.257	0.115	0.584	0.42	0.757	.0.198	.c	0.081	.935**	0.225	0.163	0.668	0.683	0.12	.892*	.912*	0.505	0.423	0.793	0.509	1

^**^ Correlation is significant at the 0.01 level (2-tailed)

^*^ Correlation is significant at the 0.05 level (2-tailed)

c. Cannot be computed because at least one of the variables is constant

## Discussion

All three ponds exceed standards for parameters except temperature which is primarily due to the addition of salt and chemicals to the pond from domestic sewage and commercial drainages entering the wastewater pond. Alkalinity was above the permissible range in some of the 32 samples. The pH values increased during the day due to the consumption of CO_2_ by algae during photosynthesis. Conversely, the release of CO_2_ during the night by algae will decrease pH values. The increase in conductivity is due to the salts and minerals carried out from the sewage adjacent to the wastewater pond. The mean of the samples indicates that DO was within acceptable limits. However, in certain sites it was found to be in a range of 0.3 to 2.1, indicating that these ponds are rich with organic matter, where the bacteria present utilize oxygen for biodegradation,^33^ indicating the presence of organic pollutants.^34^

Meanwhile, sampling results from the three ponds revealed that DO was increased (Pond 1 < Pond 2 < Pond 3), indicating the effective treatment of organic pollutants at each stage.

In borewell samples, among the four variables, pH, DO and temperature were within acceptable limits. However, the EC of all three samples exceeded acceptable limits. The fact that ground water is rich in mineral salts may explain the increase in EC.

According to Table 4, aluminum (Al), beryllium (Be), cobalt (Co), copper (Cu), lithium (Li), manganese (Mn), nickel (Ni), lead (Pb), tin (Sn) and zinc (Zn) concentrations in most of the wastewater samples collected from Pond 1, Pond 2, and Pond 3 were within permissible limits. The results of magnesium (Mg), sodium (Na), calcium (Ca), phosphorus (P), silver (Ag), arsenic (As), boron (B), barium (Ba), cadmium (Cd), chromium (Cr), molybdenum (Mo), potassium (K), antimony (Sb), and selenium (Se) concentrations in wastewater samples collected from the three ponds exceeded permissible limits. The presence of Ag might result from small-scale photography, household products such as wood polish and from domestic water treatment devices.^35^,^36^ In urban effluents and sewage sludge, As is present as dimethyl arsenic acid (DMAA) and as arsenite (As (III)).^37^ In the present case, As might have originated from household products such as washing products, medicines, garden products, wood preservatives, old paints and pigments.^38^ The presence of Cu might result from corrosion and leaching of plumbing, fungicides (copper (II) chloride), pigments, wood preservatives and antifouling paints.^21^ As a potentially toxic metalloid, Se is found in urban waste waters in low concentrations.^39^ Selenium comes from food products, food supplements, shampoos, cosmetics, old paints, and pigments.^25^ The remainder of the elements might originate from the small-scale industries whose function are unrecognized/unknown located around the vicinity of Taiz city.

Calcium, Mg, Ag, B, Ba, Cd and Cr exceeded permissible limits in borewell water samples. There are a few reasons for the increase in concentrations of these elements. First, there may not be a proper lining to the base of the wastewater/stabilizing pond. Improper lining can lead to the percolation of wastewater, thereby contaminating the aquifer or the groundwater channels. Second, wastewater is used for agriculture purposes around Taiz city. The use of wastewater rich in heavy metals can also lead to contamination of the water table through percolation. The third possibility involves the geology of the area. Beneath the ground surface, mineral or heavy metal-bearing rocks could also be a cause of increased concentrations of these elements in bore well water. The consumption of borewell water for drinking purposes might present a hazard to human health.^40^,^41^ To understand the relationship between wastewater and borewell water, Pearson correlation was performed.

### Correlation test

The results of the correlation analysis between the wastewater and borewell water samples are given in Table 6. Correlation analyses for the results obtained using ICP-OES were performed to understand the relationship between wastewater and borewell water samples. Correlation analysis is employed to resolve the degree of the linear relationship between two variables, with a range between −1 to 1. If the values obtained are closer to 1 or −1, this indicates that there is a strong positive linear relationship between the correlated variables. If the values are nearer to 0, this indicates that there is no linear relationship between the two variables. The present analysis indicated a strong correlation between wastewater and borewell water samples at a 0.01 and 0.05 level of significance. The analysis demonstrates the presence of a relationship among all of the elements/heavy metals present in wastewater and borewell water, suggesting that the elements/heavy metals present in wastewater might have percolated through the soil beds and might have reached the groundwater table.

This could have led to the presence/increase of some parameters in borewell water, thereby making it unsafe for domestic purposes.

Some heavy metals play important roles in the body's physiological and biochemical processes, while others can be toxic to humans.^42^,^43^ Continued use of groundwater from the borewells around Al-Buraihi stations might lead to acute or chronic toxicity in humans and animals. In the interest of public health, regulatory authorities should adequately handle the wastewater at Al-Buraihi station so that contamination of groundwater and soil is reduced. However, due to Yemen's civil war, this will not be possible in the foreseeable future.

## Conclusions

Systematic analyses of wastewater and borewell water samples were performed. The ICP-OES analysis indicated that pH, EC, DO and temperature and elements such as Ag, As, Al, Ba, B, Cd, Cr, iron (Fe), Mo, Ni, Se and Zn exceeded permissible limits recommended by international standards. Similarly, some of the elements exceeded permissible limits in borewell water samples. The results indicate a strong relationship between the elements present in wastewater and borewell water. The use of wastewater as a source of nutrients for irrigation has become common practice in Yemen, especially in Taiz, because of the water crisis in this city. The results of the present study showed that the treated sewage wastewater in Taiz city, Yemen is not suitable for irrigation as the elements/heavy metals get accumulated in soil/plants and could become accumulated in humans and animals through bio-accumulation. Finally, these heavy metals could reach the water table and aquifers through percolation, thereby polluting groundwater quality.
